# Quantitative real-time PCR study on persistence of pDNA vaccine pVax-Hsp60 TM814 in beef muscles

**DOI:** 10.1186/1479-0556-6-11

**Published:** 2008-09-02

**Authors:** Petr Orság, Veronika Kvardová, Milan Raška, Andrew D Miller, Miroslav Ledvina, Jaroslav Turánek

**Affiliations:** 1 Veterinary Research Institute, Department of Immunology, Brno, Czech Republic; 2 Palacky University, Faculty of Medicine and Dentistry, Department of Immunology, Olomouc, Czech Republic; 3 Imperial College Genetic Therapies Centre, Department of Chemistry, Imperial College London, London, SW7 2AZ, UK; 4 The Institute of Organic Chemistry and Biochemistry, Prague, Czech Republic

## Abstract

**Background:**

Application of plasmid DNA for immunization of food-producing animals established new standards of food safety. The addition of foreign products e.g. pDNA into the food chain should be carefully examined to ensure that neither livestock animals nor consumers develop unpredicted or undesirable side-effects.

**Methods:**

A quantitative real-time PCR (QRTPCR) methodology was developed to study the biodistribution and persistence of plasmid DNA vaccine pDNAX (pVAX-Hsp60 TM814) in mice and beef cattle. The linear quantification range and the sensitivity of the method was found to be 10 – 10^9 ^copies per reaction (500 ng/gDNA) and 3 copies per reaction, respectively.

**Results:**

Persistence of pDNAX in mice muscle tissue was restricted to injection site and the amount of pDNAX showed delivery formulation dependent (naked pDNA, electroporation, cationic liposome complexes) and mouse age-dependent clearance form injection site but pDNAX was still detectable even after 365 days. The QRTPCR analysis of various muscle tissue samples of vaccinated beef bulls performed 242–292 days after the last revaccination proved that residual pDNAX was found only in the injection site. The highest plasmid levels (up to 290 copies per reaction) were detected in the pDNAX:CDAN/DOPE group similarly to mice model. No pDNA was detected in the samples from distant muscles and draining lymph nodes.

**Conclusion:**

Quantitative real-time PCR (QRTPCR) assay was developed to assess the residual pDNA vaccine pVAX-Hsp60 TM814 in mice and beef cattle. In beef cattle, ultra low residual level of pDNA vaccine was only found at the injection site. According to rough estimation, consumption of muscles from the injection site represents almost an undetectable intake of pDNA (400 fg/g muscle tissue) for consumers. Residual plasmid in native state will hardly be found at measurable level following further meat processing. This study brings supportive data for animal and food safety and hence for further approval of pDNA vaccine field trials.

## Background

DNA-based vaccines represent a new and rapidly progressing area in vaccinology. So far, plasmid DNA (pDNA) vaccines have been reported to induce protective immunity in numerous animal models of parasitic, viral and bacterial diseases [1]. Moreover, pDNA vaccines appear to be well tolerated and exhibit a minimal risk of *in vivo *genome integration [2-8]. In addition, persistent plasmid does not replicate inside the cells [7] and there are no significant increases in anti-DNA antibodies leading to autoimmune reactions [9]. Although preclinical studies on animal models document overall safety, some issues and potential risks related to food-producing animals need to be addressed directly on target species since these represent separate issues to clinical applications. Thus far, data on the rates of clearance, or conversely persistence, of pDNA post injection into animals is only limited, therefore potential risks must be extrapolated from model animal studies. Quantitative biodistribution studies have been performed in mice [3-7,9-17], rats [18], rabbits [2,8,9,13,19], sheep [20], dog [21] and macaques [22], all post intramuscular (i.m.) administration of pDNA. Gratifyingly, all the studies have given evidence for overall safety as well.

Quantitative real-time PCR (QRTPCR) is the most widely used method for specific quantitative assay of ultra low concentration of pDNA in biological materials. Such data are necessary for the assessment of the risk of residual plasmid presence in consumable parts of DNA vaccinated livestock, mainly in muscles. Nowadays, there are no definitive guidelines available to approve usage of DNA vaccines in food- producing animals. In this work, the QRTPCR method was used for the study of the persistence of pDNA at the injection sites in mice and beef cattle. For this reason we developed an isolation and detection QRTPCR based methodology for the accurate quantification of residual levels of vaccine pDNAX (pVAX-Hsp60 TM814) in the muscles after various approaches to vaccine application (naked pDNA, pDNA with electroporation, pDNA complexed with cationic liposomes). The primary motivation for this study was to obtain data for further negotiations with the State Veterinary Authority (Czech Republic) to get the approval for field trials with pDNAX against ringworm (*Trichophyton mentagrophytes*)[23].

## Materials and methods

### Plasmids

The plasmid pDNAX (pVAX-Hsp60 TM814), encoding the heat shock protein 60 (Hsp60) from *Trichophyton mentagrophytes *[24] and the plasmid pLacZ (pcDNA3.1/LacZ), expressing β-galactosidase, were used in this study. The plasmid DNA was produced in XL-1 Blue *E. coli *strain and purified with Qiagen Giga prep kit (Qiagen, Germany) to provide endotoxin free plasmid. Plasmid integrity was confirmed by electrophoresis on 0.8% agarose gel. The UV absorbance was used for quantification of DNA (A_260_) and purity (A_260_/_280_) of plasmid preparation. The concentration of stock plasmid preparation was 2 mg/ml, the content of supercoil form was more than 90%, and the A_260_/A_280 _was between 1.8–1.90.

### Preparation of liposomes and pDNAX-liposome complex

Positively charged lipid *N*^1^-cholesteryloxycarbonyl-3,7-diazanonan-1,9-diamine (CDAN) and neutral colipid dioleoyl L-α-phosphatidylethanolamine (DOPE) in 1:1 molar ratio were used for preparation of liposomes. Fluorescently labelled liposomes were prepared by addition of 1,2-dioleoyl-sn-glycero-3-phosphoethanolamine-N-lissamine rhodamine B (PE-rd)(1 mol % of total lipids). Lipids used in this study were purchased from Avanti Polar Lipids, Inc., USA. The lipid mixture was dissolved in freshly distilled chloroform and the solvent was evaporated under reduced pressure using rotary evaporator Laborota 4000 (Heidolph, Germany). Dry lipid film was hydrated in 4 mM HEPES buffer pH 7.2. Monodisperse liposomal preparation was obtained by extrusion through 100 nm Isopore filters (Millipore, Czech Republic). The size distribution and the zeta potential of resulting liposomes were measured using Zetasizer Nano ZS (Malvern, UK). Complexes of pDNAX with liposomes were prepared by incubation of the mixture of DNA with liposomes in 1:5 weight ratio at room temperature for 20 min [25].

### pDNA application to mice

The vaccination experiments were approved by the Ethical Committee of the Veterinary Research Institute, Brno, Czech Republic.

#### Experiment I

BALB/c mice (7–8 weeks of age) were divided into one control and three test groups. Various formulations of pDNAX (naked pDNAX, naked pDNAX followed by electroporation, liposomal complex pDNAX:CDAN/DOPE) were applied by i.m. injection route. On day 0, the tested animals received single injection into the right calf muscle. In each experimental group, pDNAX (10 μg comprising approximately 10^12^-10^13 ^copies) in a total volume of 50 μl was applied. An electroporator (developed in the laboratory of Prof. Yuhong Xu at Shanghai Jiao Tong University, Shanghai) was used in these experiments. Six electric pulses (duration 20 ms, field strength 150 V/cm, the interval between the pulses 1 s, the gap distance between electrodes 3 mm) were applied by two parallel needle electrodes (distance of the needles was 3 mm) immediately after i.m. injection. Injection point was in the middle between the electrodes. 50 μl of PBS were applied to mice of the control group. The animals were kept under standard conditions during the whole experimental period. Neither lost of weight nor pathological changes in the skin, somatomotoric activity or behaviour pattern were observed. At the end of each experimental period i.e.: 1, 7, 28, 90, 180 and 365 days after administration, 4 animals from each test group and 2 animals from the control group were sacrificed. Both quadriceps muscles from each mouse were collected for the evaluation of the persistence of pDNAX. The samples of muscles were homogenised, weighted, frozen in liquid nitrogen and stored at -70°C until further processing.

#### Experiment II

The influence of the age of the mice on the dynamics of plasmid clearance during 1 month period after administration was tested on BALB/c mice 5 weeks of age. Experimental design was the same as in Experiment I.

#### Experiment III – fluorescent liposomes and analysis of gene expression

Single dose of pLacZ (10 μg) was injected into calf muscle of BALB/c mice (5 weeks of age). Plasmid pLacZ was delivered in the following forms: naked DNA, naked DNA followed by electroporation, and pDNA complexed with fluorescent cationic liposomes (CDAN/DOPE/PE-rd). The samples of muscles were taken at the day 1, 7, 14 and 28 after the administration. Tissue sections of the thickness 7 μm were prepared by cryocat Leica CM1900 (Leica, Germany) and stained for β-galactosidase expression using the substrate X-gal (Sigma, Czech Republic). The distribution and persistence of fluorescently labelled pDNA:(CDAN/DOPE/PE-rd) complexes were evaluated using fluorescence microscope Eclipse TM200 with CCD camera (Nikon, Japan) and the images were recorded using Lucia software (Laboratory Imaging Ltd., Czech Republic).

### pDNA application to beef cattle

The vaccination experiment was approved by the Ethical Committee of the Veterinary Research Institute, Brno and University of Palacky, Medicinal Faculty, Olomouc. Ten beef cattle bulls (3 months of age) were divided into three experimental groups. In each experimental group, pDNAX (500 μg per dose; this dose was found to be sufficient for induction of the immune response in calves [23]) in various formulations (naked pDNAX, pDNAX in combination with liposomal adjuvant B30-norAbu-MDP (lipophilic derivative of muramyl dipeptide entrapped into liposomes; this compound was synthetised at the Institute of Organic Chemistry and Biochemistry, Prague), complex pDNAX:CDAN/DOPE) was administered by i.m. single needle injection into right coccygeus muscle. The animals were re-vaccinated after three weeks by the same dose, formulation, and procedure. The bulls were slaughtered 242–292 days after the second vaccination and whole right coccygeus muscle (injection site), whole left coccygeus muscle (opposite-to-injection site), random tissue samples from gluteus muscle (distant muscle tissue), and poplitheal lymph nodes were collected. The samples of muscles were cut into small pieces, homogenised by blender and stored at -70°C before further processing. Various numbers of samples from particular tissues were prepared and taken for analyses: injection site (n = 5), opposite-to-injection site (n = 4), distant muscle tissue (n = 3), each draining lymph node (n = 2).

### DNA extraction from tissue sample

The isolation of genomic DNA (gDNA) from the samples of tissue taken from mice or beef cattle was performed by modification of guanidine thiocyanate (GuSCN) lysis method followed by binding of DNA to SiO_2 _[26]. The average weights of mice muscle samples and the samples from beef cattle muscles were 100–150 mg and 200 mg, respectively. The samples were mixed in 2-ml tubes with 1 ml of lysis buffer (5 M GuSCN; 0.05 M Tris-HCL, pH 6.4; 0.02 M EDTA, pH 8.0; 1.3% Triton X-100) and about 10 pcs. of 2.5 mm glass beads. The mixture was homogenised twice in Magnalyser (Roche, Germany) for 30 s at 6000 rpm. Then the suspension was centrifuged (14000 g, 10 min.); 1 ml of the supernatant from mice tissue samples or 700 μl of the supernatant from beef cattle tissue samples was transferred in 1.5 ml tube, filled with lysis buffer to the total volume of 1.2 ml, and then 50 μl of silica suspension (freshly prepared on the preceding day by mixing 100 mg of Celite with 500 μl of water and 5 μl of 32% HCl) was added. The tubes were vortexed for 30 s. The mixture was incubated at room temperature for 10 min., centrifuged (14000 g, 1 min.), and the supernatant was discarded. The silica pellet was washed twice with 1 ml of washing buffer (5 M GuSCN; 0.05 M Tris-HCL, pH 6.4; 0.02 M EDTA, pH 8.0), twice with 1 ml of 70% ethanol, and once with 1 ml of acetone. Subsequently, silica pellet was dried in heated block at 56°C for 15 min, followed by extraction step performed twice: mixing with 80 μl of tempered (56°C) TE-buffer (10 mM Tris-HCl, 1 mM EDTA pH 8.0), incubation in heated block for 10 min., and centrifugation (14000 g, 1 min.). 80 μl of the recovered supernatant was transferred into clean tube, centrifuged again (14000 g, 1 min.), and used for QRTPCR analysis. 20-μl volumes were taken from each extracted DNA sample to measure DNA concentration (A_260_), purity (A_260_/A_280_), and integrity (0.6% agarose gel electrophoresis).

### QRTPCR analysis

The Genecompare software (Applied-Maths, Belgium) was used to design primers amplifying a sequence stretch that contains plasmid specific promoter sequence (CMV) as well as sequence from *hsp60 *gene, generating 161 bp specific product. 500 ng of genomic DNA (gDNA) template was amplified in duplicate in glass capillaries in a final volume of 20 μl using 2× Real time PCR Syber green master mix (Qiagen, Germany) with 0.5 μM primers: CMV-Hsp60-F: 5'-ACTATAGGGAGACCCAAGCT-3' CMV-Hsp60 R: 5'-GCCTGTAGGTACTCGACAAC-3' Optimal PCR cycling conditions were: 15 min. pre-incubation at 95°C, 45 amplification cycles consisting of denaturation at 95°C for 10 s, annealing at 61°C for 25 s, extension at 72°C for 10 s and data acquisition at 78°C for 1 s using a temperature transition rate of 20°C/s in the LightCycler 1.5 instrument (Roche, Germany). Second derivative maximum method was used for Ct calculation from amplification curves. The amount of pDNAX in the tested samples was calculated by the comparison of the sample's Ct value with Ct values of the titration curve of genomic samples artificially spiked with pDNAX. The results for each mouse group were recalculated and are expressed as mean plasmid copy number per μg of gDNA (PCN/μg gDNA).

### Precautions to prevent contamination

All the manipulations with stock plasmid, tissue sampling, QRTPCR set up and template addition were done in separated working areas [27]. To prevent cross-contamination, the non-treated animals were handled before the vaccinated animals. Samples from the vaccinated animals were processed in the following manner: distant muscle tissues (beef cattle), muscle tissue from opposite-to-injection site (mice: left calf muscle, beef cattle: left coccygeus muscle), injection site (mice: right calf muscle, beef cattle: right coccygeus muscle). Disposable materials were used whenever possible. The work surfaces and equipment were decontaminated by either 10% bleach or DNAoff (Fluka, Germany).

## Results

### Validation of QRTPCR method

**Figure 1:**
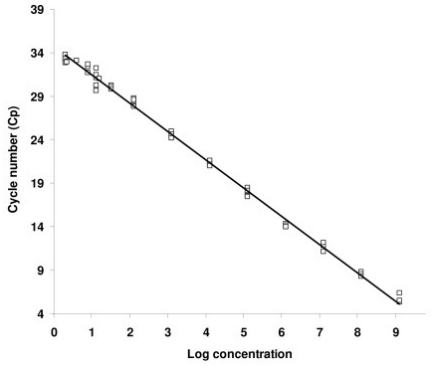
**Linearity analysis after QRTPCR amplification**. Dilution series of pDNAX (10^9 ^– 3 × 10°copies) was amplified with 500 ng of mouse gDNA. Full squares represent Cp values (crossing point) recorded from three independent pDNAX dilutions. The strait line represents linear regression analysis with correlation coefficient (R^2^) greater than 0,99.

Persistence of pDNAX was determined by a QRTPCR methodology designed to specifically recognize the stretch of promoter-insert from the pDNAX plasmid. The methodology was initially investigated for sensitivity, specificity and linearity, in the detection of pDNAX plasmid. Firstly, the detection method was studied as part of the protocol for isolation of genomic DNA (gDNA) from mouse and beef muscle tissue. This protocol for isolation was found to be scalable up to 200 mg of muscle tissue, and in repeated applications of the QRTPCR methodology no inhibition due to sample matrix or presence of inhibitors was observed. Thereafter, pDNAX was introduced to gDNA allowing the detection limit (DL) and linear quantification range (LQL) of the QRTPCR methodology to be determined. In this instance, the LQL was found to be within the range of 40-4 × 10^9 ^ag (10-1 × 10^9 ^PCN/500 ng gDNA and the DL was shown to be 10 ag (3 PCN/500 ng gDNA) (Fig. 1). Finally, mouse and beef muscle tissue samples were spiked with quantities of pDNAX in the range from 10-4 × 10^9 ^ag. Thereafter, complete pDNAX isolation procedures were performed demonstrating that pDNA recovery was in the range of 65–95%. The detection limit of pDNAX isolation from tissue samples was found to be 800 ag (100 PCN/500 ng gDNA). This parameter represents the lowest amount of pDNAX that could be detected in all replicates of spiked samples by QRTPCR.

### Biodistribution and persistence in mice

#### Experiment I

**Figure 2:**
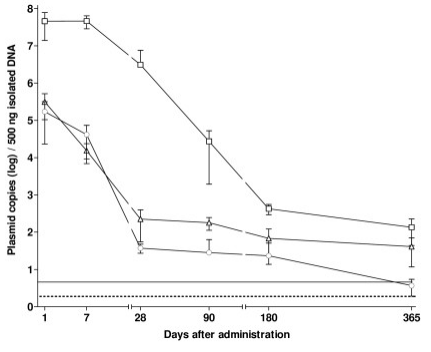
**Levels of pDNAX detected by QRTPCR in calf muscle (at the injection site) after administration of 10 μg pDNAX in 8-week old BalB/C mice**. The line connects the average levels of plasmid DNA detected by QRT-PCR in 500 ng of isolated DNA (MC/r) ± SD (four mice per time point). The straight line represents quantification limit of QRTPCR assay (10 pDNAX copies/reaction). The dotted line represent detection limit of QRTPCR assay (3 pDNAX copies/reaction). The data from control group were omitted (all control animals were negative). Routes of application: full circle denotes naked pDNAX; full triangle denotes pDNAX plus electroporation; full square denotes pDNAX:CDAN-DOPE complex.

The pDNAX plasmid (10 μg) was injected i.m. to 8-week-old mice and then detectable levels of plasmid were assayed as a function of time by QRTPCR. As shown (Fig. 2), pDNAX introduced i.m. to 8-week-old mice persisted at detectable levels in the region of the injection site for up to one year after administration regardless of the plasmid formulation and method of application. However, rates of clearance of pDNAX varied with the mode of administration. One day post injection, pDNAX remaining in muscle samples from three different groups was in the following order: pDNAX:CDAN/DOPE: 374 ng/μg gDNA (4.60 × 10^7 ^PCN/500 ng gDNA) > pDNAX electroporation: 2600 pg/μg gDNA (3.20 × 10^5 ^PCN/500 ng gDNA) > naked pDNAX: 689 pg/μg gDNA (1.70 × 10^5 ^PCN/500 ng gDNA). In the first group, pDNAX was injected in complex with CDAN/DOPE cationic liposomes; in the second group, pDNAX was injected with electroporation; in the third group naked pDNAX was injected alone. Thereafter, in the case of the pDNAX:CDAN/DOPE group levels of pDNAX were found to undergo a 10-fold decline between the day 7 and the day 28, followed by a further 100-fold decline by the day 90, so that by the day 365 a detectable level of only 535 ag/μg gDNA (1.35 × 10^2 ^PCN/500 ng gDNA) was determined by QRTPCR (Fig. 2). By contrast, in the case of both pDNAX electroporation and naked pDNAX groups, clearance rates were more considerable. In the case of the naked DNAX group, final plasmid levels were found to be below the quantification limit of 40 ag/μg gDNA (10 PCN/500 ng gDNA (Fig. 2).

#### Experiment II

**Figure 3:**
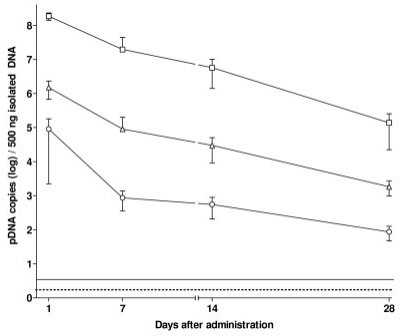
**Levels of pDNAX detected by QRTPCR in calf muscle (at the injection site) after administration of 10 μg pDNAX in 5-week old BalB/C mice**. The line connects the average levels of plasmid DNA expressed in logarithm scale detected by QRTPCR in 500 ng of isolated DNA (MC/r) ± SD (four mice per time point). The straight line represent quantification limit of QRTPCR assay (10 pDNAX copies/reaction). The dotted line represent detection limit of QRTPCR assay (3 pDNAX copies/reaction). The data from control group were omitted (all control animals were negative). Routes of application: full circle denotes naked pDNAX; full triangle denotes pDNAX plus electroporation; full square denotes pDNAX:CDAN-DOPE complex.

Identical experiment was performed with 5-week-old mice to evaluate a possible relationship between the animal age and the rate of clearance of pDNAX from the site of injection. In both cases, naked pDNAX and pDNAX electroporation groups, the rates of clearance of pDNAX were found to be slower for 5-week-old mice in comparison to the corresponding situation in 8-week-old mice (compare Fig. 2 and Fig. 3). Nevertheless, the final differences in pDNAX levels between pDNAX:CDAN/DOPE and the pDNAX electroporation groups were still in the range of 100-fold, with an even greater gap of over 10^4^-fold between pDNAX:CDAN/DOPE and naked pDNAX groups. In this instance too, a difference of 1–2 orders of magnitude also existed between the measured plasmid levels in the pDNAX electroporation group and the naked pDNAX group at all time points analyzed (Fig. 3), in partial contrast to our observations with 8-week animals (Fig. 2).

#### Experiment III- analysis of gene expression and distribution of fluorescent complex of pDNA/cationic liposomes

**Figure 4:**
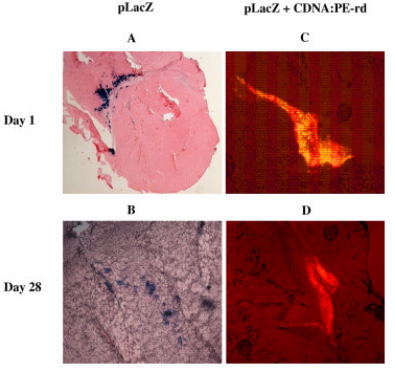
**Expression of β-galactosidase activity and persistence of fluorescent liposome- pLacZ complexes in mice calf muscles**. Mice calf muscles were histochemically stained for β-galactosidase activity at the day 1 (A) and at the day 28 (B) after i.m. injection of 10 μg pLacZ followed by electroporation. Histological detection of fluorescent liposome-pDNA complex (10 μg pLacZ/CDAN:PE-rh) in mice calf muscles at the day 1 (C) and 28 (D) after administration into young mice (the age of 5 weeks).

Flourescently labelled pDNAX:CDAN/DOPE complexes were prepared and injected i.m. into 5-week old mice in order to make comparison with the QRTPCR data (Fig. 3). Post administration, complexes were clearly visible, localised at the site of application, and persisted for more than four weeks as shown in histological sections by fluorescent microscopy (Fig. 4). This is in a good correlation with the persistence of pDNAX as determined by QRTPCR (Fig. 3). Similar data were found in the group of 8-week-old mice (data not shown). Transfection experiments were then performed by the administration of naked pLacZ injected *i.m*. into 5-week and 8-week old mice. Histological analyses of muscle tissue sections revealed that β-galactosidase expression was undetectable after the injection to 8-week old mice with naked pLacZ (10 μg) (data not shown). However, when pLacZ (10 μg) was introduced together with electroporation pulse, transfection was detectable, but only a few myocytes were found to be positive for β-galactosidase expression. In contrast, β-galactosidase expression was much more evident with 5-week old mice. Myocyte bundles expressing β-galactosidase were clearly localised around the site of injection and there was little tissue damage associated with electroporation. Several β-galactosidase positive myocytes were found also four weeks after electroporation. Micrographs of the tissue sections documenting β-galactosidase expression are presented (Fig. 4).

### Biodistribution and persistence in beef cattle

Residual pDNAX levels in various samples of tissues taken from beef cattle slaughtered 9 months after application of plasmid are summarized (Table 1). QRTPCR examinations of muscle tissue taken from the injection site revealed very low residual or nearly zero pDNAX levels in all animals tested. Plasmid levels detected in animals injected with naked pDNAX group were predominantly below quantification 40 ag/μg gDNA (10 PCN/500 ng gDNA) or detection 13 ag/μg gDNA (3 PCN/500 ng gDNA) limit. Slightly higher residual plasmid levels, but mostly close to quantification limit, were also detected in the cases where pDNAX was injected with a liposomal formulation of adjuvant B30-norAbu-MDP. The highest levels of retention (288 PCN/500 ng gDNA) were recorded at the injection site in the muscle samples from beef cattle injected with pDNAX:CDAN/DOPE. However, plasmid levels from all slaughtered animals showed progressive decreases in pDNAX levels below the quantification limit after longer time periods. Gratifyingly, essentially no plasmid was found at either distant muscle tissue or in draining lymph node samples. Muscle samples from opposite-to-injection site (internal negative control) were also negative for the presence of pDNAX.

**Table 1:** Effect of various pDNAX formulations on its persistence in beef cattle after i.m. administration

Beef cattle groups	Beef cattle ID code	Interval between 2^nd ^immunisation and slaughter (days)	pDNA copies at the injection site/500 μg DNA (n = 5)	pDNA copies opposite -to- injection site muscle (n = 4)	pDNA copies distant muscle (n = 3)	pDNA copies DLN^a ^total (n = 6)
pDNA	20087	242	< LQL (2); < DL (2); Neg. (1)	0/4	0/3	0/6
	20105	277	73.97; 35.59; < LQL (2); < DL (1)	0/4	0/3	0/6
	20080	284	< LQL (5)	0/4	0/3	0/6

DNA + B30-Nor-AbuMDP	20083	242	19.86; 16.23; 15.5; < LQL (2)	0/4	0/3	0/6
	28504	270	92.78; 29.25; 28;92; 24.04; 23.75	0/4	0/3	0/6
	20090	291	13.48; 12.73; 10.87; < LQL (1); Neg. (1)	< DL(1/4)	0/3	0/6

DNA:cationic liposome complex	20086	270	288; 220.08; 200.60; 30.07; < LQL (1)	0/4	0/3	0/6
	20089	277	228.9; 169.90, 134.70; 39.57; 39.00	0/4	0/3	0/6
	3654	291	149.3; 64.79; 46.60; 18.88; 18.73	0/4	0/3	0/6
	20081	298	< LQL (5)	0/4	0/3	0/6

The total amount 1000 μg of pDNAX in two equal doses was delivered into coccygeus muscle (injection site) as naked pDNAX, naked pDNAX + liposomal B30-norAbuMDP, and cationic liposome complex pDNAX:CDAN-DOPE. The level of pDNAX in the injection site, opposite-to-injection site, distant muscle tissues and draining lymph nodes was examined after 242–298 days after the second immunisation. Plasmid copies are expressed as mean plasmid copies per 500 ng of genomic DNA (MC/r) from duplicate QRTPCR assay. < LQL: below linear limit of quantification (10 copies/reaction), < DL: below detection limit (3 copies/reaction), Neg.: negative sample, ^a^DLN- draining lymph nodes.

### General safety

After the injection of pDNAX (or pLacZ), both mice and cows from all the tested groups survived throughout the duration of the experiments and neither any apparent pathological changes at the site of injection nor loss of body weight were observed indicating that pDNAX (pVAX-Hsp60 TM814) vaccine and its formulations as a complex with cationic liposomes or liposomal adjuvant B30-norAbu-MDP were well tolerated in both species. Application of electroporation with or without previous local or general anesthesia did not lead to any changes of somatomotoric activity or even paraplegia in mice.

## Discussion

Limited data on the examination of the effect of pDNA vaccines on food-producing animals have been reported so far and we can only extrapolate the results obtained in the model animals. Different regulation acts on genetically modified organisms and their interpretation by national authorities represent serious obstacles for the field of DNA vaccination experiments on large animals. DNA vaccines have not yet been licensed in many countries, therefore national authorities are not experienced with this kind of product and do not differentiate between gene medication and gene modification. Within the EU, two opposite points of view are maintained as regards DNA vaccinated animals. The first one, held by The British Agriculture and Environment Biotechnology Committee, does not consider DNA vaccinated animals as genetically modified ones due to the low risk of insertion of pDNA into genome. The second one, held by The Norwegian Directorate for Nature Management, states that DNA vaccinated animals should be considered as genetically modified for as long as the added DNA is present. In other words, gene medication is the subset of gene modification [28]. The safety concerns raised by the use of plasmid DNA for immunization of food producing animals, livestock and poultry are obviously distinct from those in humans. The addition of foreign products e.g. pDNA into the food chain should be carefully considered to ensure that neither livestock animals nor consumers develop unpredicted or undesirable side-effects. While the safety of DNA vaccines was documented in animal and human trials, the problem of residual plasmid in consumable parts of livestock and poultry has not yet been solved on the level of the State Veterinary Authority and regulatory veterinarians. In contrast to experiments performed on small rodents, vaccination field trials on large animals, e.g. cows, are more expensive and are subjected to more strict regulations.

The condemnation of whole animals and the processing of their cadavers in rendering plants pose not only an economic problem but also an ethic one. The presented study has shown that pVAX-Hsp60 TM814 vaccine and its formulations as a complex with cationic liposomes or liposomal adjuvant B30-norAbu-MDP were well tolerated by both species. From the practical point of view, the regulatory authorities will demand a reliable, sensitive and cost effective method for the determination of the amount of residual plasmid and its localization in the body at the time of the slaughter. The detection method based on QRTPCR was proved to be suitable for the exact quantification of residual plasmid levels in muscle tissues after i.m. application of pDNA vaccine. By the use of the artificially spiked muscle tissue samples we documented, that pDNA was efficiently recovered (65–95% of the initial amount) within the wide range of plasmid concentrations that might occur in real tested samples (Fig. 1). The quality of the isolated DNA was sufficient for the development of QRTPCR assay providing parameters ensuring high specificity, sensitivity and reproducibility for the precise pDNA quantification. The sensitivity of our assay was comparable to that published by Tuomela for the pDNA GTU^®^-MultiHIV [18].

### Biodistribution and persistence of pDNA in mice

Model studies on rodents covering overall biodistribution and safety features are required before DNA vaccines enter human clinical trials [29]. We used mouse model to provide information about plasmid clearance kinetics, which is useful for further extrapolation for beef cattle. Biodistribution studies, primarily those performed with naked pDNA applied i.m., show that pDNA is completely cleared from the injection site within 28 days or even sooner. However, long-term persistence was reported as well – by qualitative PCR: 18 wks [11], 180 days [7], 19 months [10], and 2 years [16] after application.

Our results confirm the previous observations that plasmid DNA is rapidly cleared from the injection site [15,17,30]. Depending on the type of application, the amount of pDNA found in mice after 24 hours in electroporated and naked group was less than 0.1% and less than 0.01%, respectively. Naked pDNA is immediately subjected to degradation, therefore only limited fraction of the applied plasmid is capable to reach the zone where pDNA is protected (i.e. structures like T-tubules and caveolae [31]), against the attack of serum and tissue specific nucleases [32].

Application of electroporation pulse leads to transient membrane disruption facilitating pDNA uptake. Generally, electroporation improves pDNA uptake and leads to several orders higher expression levels, as reviewed in [33]. However, for further optimization of electroporation parameters for clinical application it is necessary to reduce a pain and potential muscle damage caused by this technique [34-36]. The study published by Wang et al. [37] determined, that critical parameters influencing electroporation are plasmid concentration, injection volume, concentration of saline media, size of plasmid DNA, repeated gene transfer. However, neither the influence of lag time between plasmid injection and electroporation nor the effect of the age of mice was observed. On the contrary, we detected the age-dependent differences (5-week old mice vs. 8-week old mice, Fig. 2 vs. Fig. 3) of residual plasmid in muscles of mice vaccinated by naked pDNA or electroporated. This could be explained by the age-dependent changes of extracellular matrix structure, which might affect the permeation of pDNA and hence the efficiency of electroporation resulting in the decreased transfection efficacy in the older mice [38]. This consideration is also confirmed by our data obtained with 5-week old mice, where the differences between the naked DNA and the electroporated group were more clearly pronounced (Fig. 3) and a slower clearance rate within the first 28 days was observed (compare Fig. 2 and Fig. 3). Such important effect of extracellular matrix on local pDNA delivery was documented using the enzyme hyaluronidase that breaks down the components of extracellular matrix [39-41]. Rapid plasmid decline in naked and electroporated group within the first 28 days (Fig. 2) could be also related to transfection of other cells than myocytes, e.g. endothelial cells, in which plasmid DNA is unstable and could be lost during mitosis. Relative stability of low plasmid level in muscle was observed within the period of the day 28 and 1 year after administration. pDNA is supposed to be located in the nucleus of myocytes, which can retain pDNA for a long time. Gradual decline of pDNA concentration could be explained by normal myonuclei turn-over in myocytes [42]. For the exact evaluation, whether the plasmid is integrated into genomic DNA or presented in extrachromosomal state, a precise gel purification method would be necessary [4,5,12,13]. Furthermore, plasmid integration into genomic DNA is a very rare event, usually lower than the level of spontaneous mutation [4]. Wang et al. [5] reported that less than 0.2% of the intracellulary presented pDNA was integrated into genomic DNA after application of naked and electroporated plasmid, respectively. According to such calculations, plasmid integration into genomic DNA in our experiments would be mostly at the level below quantification limit or even undetectable.

Cationic liposomes are mostly used as carries for intravenous systemic delivery, but novel lipid combinations might be suitable for i.m. delivery [2,43,44] and they have been found to be well tolerated in both, animals and humans [45]. When we compared the cationic liposomes with the standard method of i.m. delivery, i.e. the injection of naked pDNA without or with electroporation, plasmid levels retained in mouse muscles after 24 hours from pDNA:CDAN-DOPE group were even 100–1000× higher (between 7–11% of the initial amount). Generally, our data demonstrated a slower clearance of pDNA from the injection site of pDNA:CDAN-DOPE group within the period of day 1 and day 28 in comparison to both, the naked and electroporated groups (Fig. 2 and Fig. 3). This data would support the consideration that pDNA in liposomal complex is more protected against the attack of nuclease. With regards to the observation of Hartikka et al[43], who noticed that another cationic lipid formulation – Vaxfectin did not appear to increase transfection, we can suppose that high plasmid levels are located extracellulary. Using fluorescently labelled liposomes, histological analysis revealed that liposomal complexes were mostly distributed along the injection lane, forming a depot within muscle tissues even after 28 days (Fig. 4).

### Biodistribution and persistence of pDNA in beef cattle

In order to facilitate further plasmid detection and potentially minimise a condemnation of whole consumable parts, coccygeus muscle was chosen as a suitable site for immunization. It is important to note that this small muscle, located closely to the root of the tail, is easy to reach and remove after slaughter. Having 10 animals available in experimental herd, we tested i.m. administration of pDNA vaccine and its various formulations intended for field vaccination trials. Unfortunately, we had not suitable electrodes for the electroporation of larger animals at the time of the experiments. Instead of electroporation we applied pDNA vaccine in combination with liposomal adjuvant B30-norAbu-MDP, which was proved to be effective in guinea pigs immunized by the same pDNA vaccine (unpublished results). Altogether, the performed QRTPCR assay revealed that pDNA persisted in ultra-low level at the injection site even 292 days after the second administration of pDNA. The highest amount of pDNA was detected in the group vaccinated by pDNA:cationic liposome complexes. These data are in good accordance with the results obtained in mice. The values of residual pDNA in the group injected by naked pDNA were mainly non-quantifiable. Combination of naked pDNA with the liposomal adjuvant B30-norAbu-MDP resulted in levels of residual pDNA close to quantification limit. It is important to emphasize that no plasmid was detected in distant muscle tissue, in draining lymph node or in the opposite muscle directly connected with these lymph nodes. The tissues located contralaterally to the injection sites could also be considered as negative controls for each vaccinated animal.

## Conclusion

Quantitative real-time PCR (QRTPCR) assay was developed to assess a residual pDNA vaccine pVAX-Hsp60 TM814 in mice and beef cattle. In beef cattle, ultra low residual level of pDNA vaccine was found only at the site of injection. According to rough estimation, consumption of muscles from the injection site represents almost an undetectable income of pDNA (400 fg/g muscle tissue) for the consumers. Residual plasmid in native state will hardly be found at measurable level following further meat. This study brings supportive data for animal and food safety and hence for further approval of pDNA vaccine field trials.

## Competing interests

The authors declare that they have no competing interests.

## Authors' contributions

PO carried out development of QRTPCR, participated in quantification of pDNA, and participated in preparation of the manuscript. VK participated in preparation of cationic liposomes, carried out the histology experiments and electroporation. MR designed and prepared the plasmid for vaccination and participated in preparation of the manuscript. ADM designed and synthesised cationic lipids. ML designed and synthesised muramylglycopeptide adjuvans. JT conceived of the study, participated in its design and coordination, prepared and characterised liposomes, performed immunisation experiments and drafted the manuscript. All authors read and approved the final manuscript.
